# Peer Review of “Seroprevalence of SARS-CoV-2 in Niger State: Pilot Cross-Sectional Study”

**DOI:** 10.2196/49866

**Published:** 2023-10-17

**Authors:** Ari Samaranayaka

**Affiliations:** 1 Biostatistics Centre, University of Otago, Dunedin, New Zealand

**Keywords:** COVID-19, pandemic, SARS-CoV-2, seroprevalence, serology, epidemiology, Niger State, Nigeria, COVID-19 testing, social distancing

*This is a peer-review report submitted for the paper “Seroprevalence of SARS-CoV-2 in Niger State: Pilot Cross-Sectional Study.”*

## Round 1 Review

This is a pilot study [1] to determine the COVID-19 seroprevalence, patterns, dynamics, and risk factors in Niger State, Nigeria. The study design is a cross-sectional survey using clustered, stratified random sampling over 5 days; the prevalence was measured by detecting antibodies.

Major point: the study design uses clustered, stratified random sampling. The authors haven’t described the clusters or stratification. However, I understand this as study participants were allowed to have different, but known, probabilities of being selected for the sample. This is different to study designs where participants are selected with equal probability. However, none of the analyses presented in the manuscript accounted for this different probability of selection; all the analyses have assumed an equal probability of selection. This is a fundamental mistake of the analysis. This invalidates all the results presented in the manuscript. The term “sampling weights” is not used at all.

The aims include determining the risk factors and dynamics of COVID-19. Not sure if the authors measured the dynamic of COVID-19 at all. Also, they need to say what is meant by risk factors because they haven’t measured it if a risk factor means a causative risk factor.

For the above reasons, it is unnecessary to review this manuscript further. However, some of the points I have already noticed are listed below if the authors would like to consider them.

- The justification for this pilot study is unclear. Specifically, what will be the full study that corresponds to this pilot? Since the COVID-19 situation changes rapidly, can the lessons from this study be used for designing a full study at a later stage?
- Some of the people sampled have not consented. How do they fill those gaps? Did they sample someone else in those places? What was the response rate as a measure of sampling bias in estimating prevalence?
- The inclusion and exclusion criteria are not given. The presented results are simple percentages from participants.
- The stratification is by place of residence (2 groups), gender (2 groups), occupation (unknown number of groups), and age (unknown number of groups). Therefore the number of strata should be large, although unknown to me. I wonder what could be the justification of these strata that must have resulted in a very small number of people per strata given the total sample size of 185.
- There are multiple places that require references (eg, second paragraph under section 2.4).
- Not sure what the value is of lots of bar graphs. Almost all of the information in those graphs is already in the text.
- The text needs revising in some places. For example, the first 1.5 paragraphs under section 3.2 do not belong in the Results section. Two of the subfigures in Figure 3 have been cited but mixed up in the second paragraph of that section.
- Have they considered the incubation period needed to develop antibodies when interpreting the calculated percentages as prevalence?
- Authors have determined the sensitivity and specificity as 100% for test kits; this was using the results from 15 individuals. I am skeptical to accept that in the absence of CIs.
